# Multicenter Evaluation of Geometric Accuracy of MRI Protocols Used in Experimental Stroke

**DOI:** 10.1371/journal.pone.0162545

**Published:** 2016-09-07

**Authors:** Xenios Milidonis, Ross J. Lennen, Maurits A. Jansen, Susanne Mueller, Philipp Boehm-Sturm, William M. Holmes, Emily S. Sena, Malcolm R. Macleod, Ian Marshall

**Affiliations:** 1 Centre for Clinical Brain Sciences, University of Edinburgh, Edinburgh, United Kingdom; 2 University/BHF Centre for Cardiovascular Science, Queen's Medical Research Institute, University of Edinburgh, Edinburgh, United Kingdom; 3 Edinburgh Preclinical Imaging, University of Edinburgh, Edinburgh, United Kingdom; 4 Department of Experimental Neurology, Center for Stroke Research Berlin, Charité University Medicine Berlin, Berlin, Germany; 5 Core Facility 7 T Experimental MRIs, Center for Stroke Research Berlin and Cluster of Excellence NeuroCure, Charité University Medicine Berlin, Berlin, Germany; 6 Glasgow Experimental MRI Centre, Institute of Neuroscience and Psychology, University of Glasgow, Glasgow, United Kingdom; UNITED STATES

## Abstract

It has recently been suggested that multicenter preclinical stroke studies should be carried out to improve translation from bench to bedside, but the accuracy of magnetic resonance imaging (MRI) scanners routinely used in experimental stroke has not yet been evaluated. We aimed to assess and compare geometric accuracy of preclinical scanners and examine the longitudinal stability of one scanner using a simple quality assurance (QA) protocol. Six 7 Tesla animal scanners across six different preclinical imaging centers throughout Europe were used to scan a small structural phantom and estimate linear scaling errors in all orthogonal directions and volumetric errors. Between-scanner imaging consisted of a standard sequence and each center’s preferred sequence for the assessment of infarct size in rat models of stroke. The standard sequence was also used to evaluate the drift in accuracy of the worst performing scanner over a period of six months following basic gradient calibration. Scaling and volumetric errors using the standard sequence were less variable than corresponding errors using different stroke sequences. The errors for one scanner, estimated using the standard sequence, were very high (above 4% scaling errors for each orthogonal direction, 18.73% volumetric error). Calibration of the gradient coils in this system reduced scaling errors to within ±1.0%; these remained stable during the subsequent 6-month assessment. In conclusion, despite decades of use in experimental studies, preclinical MRI still suffers from poor and variable geometric accuracy, influenced by the use of miscalibrated systems and various types of sequences for the same purpose. For effective pooling of data in multicenter studies, centers should adopt standardized procedures for system QA and in vivo imaging.

## Introduction

Animal studies of disease models often use geometric measurements from structural magnetic resonance imaging (MRI) data as primary outcomes for evaluating the efficacy of tested interventions. Conventional techniques, such as T_1_-weighted or T_2_-weighted imaging, are employed to quantify the extent of tissue injury in stroke [1], cancer [2] and multiple sclerosis [3], among other diseases. In many cases, the developing lesion in vivo is assessed longitudinally over extensive periods of time for retrospective evaluation of treatment effects [4–6]. However, despite the current widespread use of high-field MRI scanners in preclinical research, standard quality assurance (QA) approaches for monitoring and optimizing their performance on a routine basis, similar to those normally employed in a clinical environment [7, 8], have not yet been developed. As a consequence, any drifts in scanner performance may not be picked up; this could lead to geometric inaccuracies and/or degraded image quality with direct effect on the quantification of anatomical outcomes across the duration of a study, particularly in models of neurological diseases where outcomes of interest are often very small. In addition, MRI methodology including scanning protocols and data analysis techniques vary significantly between research centers [9]. The impact of these shortfalls will become more evident in the future, as animal experimentation is set to shift from single-center to multicenter studies [10, 11]. Such studies will have high demands for accuracy, reproducibility and concordance of measurements between scanners for efficient pooling of data and valid statistical inferences, and unless the conduct of MRI is of a sufficient standard across preclinical imaging centers these requirements cannot be met.

While the accuracy and comparability of different clinical scanners in structural imaging has been intensively studied, different preclinical scanners have not yet been evaluated or compared in the same study. We set out to assess geometric accuracy of preclinical MRI systems in the context of multicenter preclinical stroke trials. As such, we chose systems routinely used in experimental stroke and devised a simple protocol for their assessment involving imaging of a small structural phantom using both standard and center-specific sequences. Moreover, within-scanner stability was evaluated at a single site over a period of six months covering time points often used in longitudinal studies of experimental focal cerebral ischaemia assessing the evolution of MRI lesions [4, 5].

## Materials and Methods

### Structural Phantom

**Fig 1:**
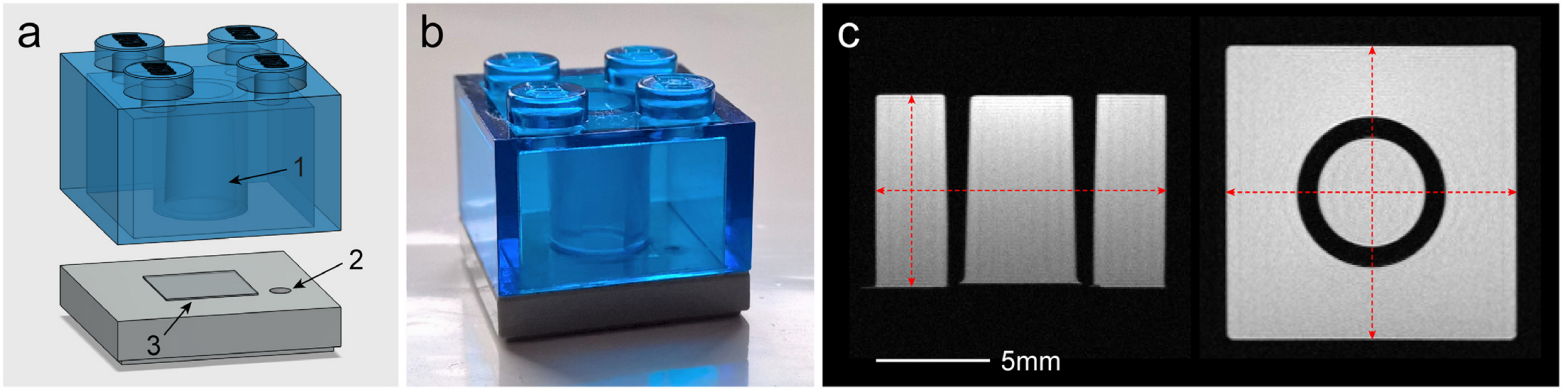
A simple structural phantom. (a) An illustration of the two plastic parts constituting the phantom, (b) a photograph of the assembled phantom and (c) sample MRI slices in the axial or sagittal (left) and coronal (right) planes through the middle of the phantom. The black arrows indicate: (1) the central compartment used for the assessment of volumetric accuracy, (2) an orientation mark carved on the base plastic and (3) transparent tape (four layers) attached to the center of the base plastic to ensure full separation between the central compartment and the rest of the phantom. The internal dimensions of the whole phantom, as shown by the dashed red arrows in (c), are measured to estimate linear scaling errors. Measurements in *y* direction (vertical in axial/sagittal slices) are obtained along the side of the phantom to avoid confounding by the tape attached to the center of the base (additional information in S1 Appendix).

A number of identical cuboid phantoms were used for scanning. They were constructed using two types of LEGO^®^ (Billund, Denmark) parts made of transparent polycarbonate and opaque acrylonitrile butadiene styrene polymers (Fig 1). These polymers have minimal water absorption properties (0.15% and 0.2–0.4% over a 24-hour period respectively [12]) and are used for the manufacturing of LEGO by injection molding with high precision molds giving the pieces an excellent geometrical tolerance of 20μm [13]. The phantoms were filled with distilled water doped with Gd-DOTA (Dotarem^®^, Guerbet, Roissy CdG Cedex, France) at 3mmol/L. This concentration was chosen according to a published table [14] to create a solution with a T_2_ relaxation time similar to that of the healthy striatum in the rat brain at 7T [15]. Using a multi-slice multi-echo sequence with 20 echo times between 10 and 200ms the corresponding T_2_ value for the phantom solution was estimated to be 53.1ms. The two parts were then permanently sealed using epoxy resin. These phantoms enable assessment of geometric accuracy in MRI scanners through measurements of their internal dimensions across all three orthogonal directions and the volume of their central cylindrical frustum-shaped compartment (Fig 1).

The dimensions of all constructed phantoms were measured before filling using calipers to verify inter-phantom precision (internal dimensions along *x*, *y* and *z* directions are 12.80mm, 8.45mm and 12.80mm respectively). To measure the true volume of the central compartment of the phantom, 3D computed tomography (CT) images of three empty phantoms were obtained using a nanoScan^®^ PET/CT scanner (Mediso Ltd., Budapest, Hungary) with an isotropic resolution of 34.5μm. The CT data were first smoothed using a Gaussian kernel (81.2μm full width at half maximum) and then binarized using an appropriate intensity threshold giving a phantom mask with exactly the caliper-measured dimensions. The volume of the central compartment in all three phantoms was measured from the thresholded CT data and the mean value was considered as the ground truth (153.14mm^3^). Analysis was performed in ImageJ (1.50b, Rasband, W.S., National Institutes of Health, Bethesda, Maryland, USA, http://imagej.nih.gov/ij/). CT images were also used as reference images for comparison with MRI data using registration methods, as described later.

### Between-Scanner Variability

Six preclinical MRI scanners located in six different centers across Europe were evaluated (labelled “A”, “B”, “C”, “D”, “E” and “F”; Table 1). All scanners except “A” were included in this study as they are routinely used for the assessment of lesion sizes in rat models of stroke in vivo. Since our aim was to observe the variability between scanners in stroke imaging specifically and not perform an absolute comparison of identical systems, each center was asked to use their standard coil set-up for brain imaging. This consisted of volume coils for radiofrequency (RF) transmission and surface coils for signal reception for all centers (Table 1). In a manner similar to the standard rodent neuroimaging procedure, the phantom was attached to the surface coil and the latter was placed on the animal cradle. This was then positioned in the isocenter of the magnet. All evaluated systems undergo only standard preventive maintenance service annually by external engineers and the gradient coils of none of them were calibrated before our experiments. In addition, no system employs a method for automatic gradient non-linearity distortion correction.

**Table 1:** Details of Scanners and Imaging Coils.

Details	A	B	C	D	E	F
**Scanner manufacturer and model**	Agilent Technologies^®^ (Varian^®^)	Bruker^®^ BioSpec^®^ 70/30	Bruker BioSpec 70/30	Magnex Scientific magnet, Bruker gradient coils	Bruker BioSpec 70/30	Bruker BioSpec 70/20
**Field strength (Tesla)**	7	7	7	7	7	7
**Magnet bore diameter (mm)**	305	300	300	160	300	200
**Maximum gradient strength (mT/m)**	400	400	600	750	200	440
**Scanner software and version**	VnmrJ^®^ 3.2	ParaVision^®^ 5.0	ParaVision 5.1	ParaVision 5.0	ParaVision 5.1	ParaVision 6.0
**Inner diameter of volume RF transmit coil (mm)**	72	72	72	72	72	86
**Type of surface RF receive coil**	Rat head 2-channel phased array	Rat head 4-channel phased array	Rat head 4-channel phased array	Rat head	Mouse head	Rat head 2-channel phased array

RF indicates radiofrequency.

The phantoms were scanned using two 2D structural sequences: a standard one based on a widely used type of imaging sequence [9] at all centers (labelled “a”; Table 2), and a center-specific in vivo sequence for the assessment of infarct size in the rat model of stroke (labelled “b”, “c”, “d”, “e” and “f”; Table 2). Scanner “A” was assessed using sequence “a” alone as no stroke experiments were performed in this center prior to this study. The phantoms were scanned six times using each sequence: once in each primary imaging plane (axial, coronal and sagittal), and all repeated with flipped frequency and phase encoding directions. Each phantom was scanned before any other imaging experiments during the day to guarantee normal system operating temperatures. To ensure further correspondence across centers, imaging adhered to a pre-prepared form with detailed instructions for phantom placement, slice positioning and scanning, as well as exemplar images.

**Table 2:** Imaging Parameters of Standard (“a”) and Stroke (“b”-“f”) Pulse Sequences.

Parameters	a	b	c	d	e	f
**Type of sequence**^a^	FSE	RARE	MSME	RARE	RARE	RARE
**TR (ms)**	1600	5000	3375	2742	3000	3500
**TE_eff_ (ms)**	20	47	11–176^b^	33	24	33
**Averages**	2	2	1	4	1	4
**Echo train length**	4	8	N/A	8	4	8
**Receive bandwidth (kHz)**	100.0	50.0	59.5	47.0	50.0	32.9
**Field of view (mm)**	19.2×19.2	25.0×25.0	19.2×19.2	40.0×40.0	25.0×25.0	25.6×25.6
**Matrix size (pixels)**	256×256	256×256	256×256	256×256	256×256	256×256
**Slice thickness^c^ (mm)**	1	0.75	1	1	0.6	0.5
**Voxel size (×10^-3^mm^3^)**	5.6	7.2	5.6	24.4	5.7	5.0
**Scanning time (min:sec)**	3:28	5:20	10:48	5:28	3:12	7:28

In vivo T_2_-weighted sequences “b”-“f” are used at corresponding centers “B”-“F” (Table 1) for assessing infarct size. FSE indicates fast-spin echo; MSME, multi-slice multi-echo; N/A, not applicable; RARE, rapid acquisition with relaxation enhancement; TE_eff_, effective echo time; TR, repetition time.

^a^RARE is the name of FSE sequence in Bruker systems.

^b^An MSME sequence uses a list of increasing echo times to quantify T_2_ relaxation.

^c^None of the sequences used an interslice gap.

### Within-Scanner Variability

Based on the between-scanner variability measurements of the internal dimensions of the phantom using standard sequence “a”, the three gradient coils of scanner “A” were calibrated by altering the strengths of the three gradient coils accordingly to minimize distortion errors along each orthogonal direction (standard procedure provided by vendor). Then, starting immediately after calibration, we performed scanning at daily, weekly and monthly intervals to assess short-term and long-term variability, corresponding to imaging over a time period of about six months. The same coil set-up was used and the same set of six scans were acquired at each time point, as described before. Each scanning session including phantom positioning lasted between 1–1.5 hours.

### Image Analysis

Raw data acquired using stroke sequence “c” were pre-processed to create quantitative T_2_ relaxation maps, in accordance with the stroke imaging protocol used in center “C” (using MRI Processor plugin in ImageJ). The maps were then inverted so that the contrast between the phantom and background is matched with that of images from all other centers and sequences. The internal dimensions of the whole phantom, approximated by the distance between opposing edges horizontally or vertically in the images, were measured in scans in all imaging planes (Fig 1c). The volume of the phantom’s central compartment was measured in axial scans alone by identifying it in all slices, counting included pixels and then multiplying by the voxel size. Both analyses were performed using a dedicated graphical tool developed in-house in MATLAB^®^ (2015a, The MathWorks Inc., Natick, Massachusetts, USA). This tool allowed fast and reproducible assessment of images and was validated against manual analysis of a set of simulated data (more information regarding its use and validation in S1 Appendix and S1 Fig). Measurements were compared with corresponding ground truth values to estimate percent linear scaling and volumetric deviations.

To aid the interpretation of geometric errors measured in the between-scanner variability assessment, 2D deformation maps showing the distance each pixel in the MRI images must move in order to recover the true shape of the phantom were created. For this, the middle slices of each MRI dataset were initially pre-processed to correct for bias field using the N4 algorithm in 3D Slicer (http://www.slicer.org [16]). Then, their mean intensity was normalized based on the intensity in the CT scans. Images acquired using stroke sequences were scaled using bicubic interpolation to ensure identical in-plane resolution for proper comparison. Subsequently, rigid transformation was used to align MRI images over corresponding reference CT data and a non-rigid b-splines transformation algorithm using the bUnwarpJ plugin in ImageJ was applied to produce a precise match [17]. This tool produces a file containing the horizontal and vertical displacement values induced on each pixel of the MRI images; this file was used to compute the Euclidean distance per pixel in MATLAB, to generate color maps indicating the extent of geometric distortion.

### Statistical Analysis

From each set of six scans we obtained 12 measurements for the phantom dimensions in total, corresponding to four measurements per orthogonal direction *x*, *y* and *z*. Linear scaling and volumetric errors were summarized using median values or ranges where appropriate. To examine how pulse sequence parameters affect volumetric accuracy in the between-scanner variability assessment, we compared measured volumetric errors with corresponding volumetric errors predicted by scaling errors alone. Each predicted error, *pe*_*v*_, was calculated using the medians of linear scaling errors across each direction (*e*_*x*_, *e*_*y*_ and *e*_*z*_)according to the formula:

$$pe_{V}=100\times \frac{\left(e_{x}+100\right)\times \left(e_{y}+100\right)\times \left(e_{z}+100\right)}{10^{6}}-100$$ (1)

SPSS^®^ (22.0, IBM Corp., Armonk, New York, USA) was used to perform a Mann-Whitney U test to examine the difference between scaling errors across the two encoding directions using all measurements from scanner’s “A” longitudinal assessment; a *p*-value less than 0.05 was considered statistically significant.

## Results

**Fig 2:**
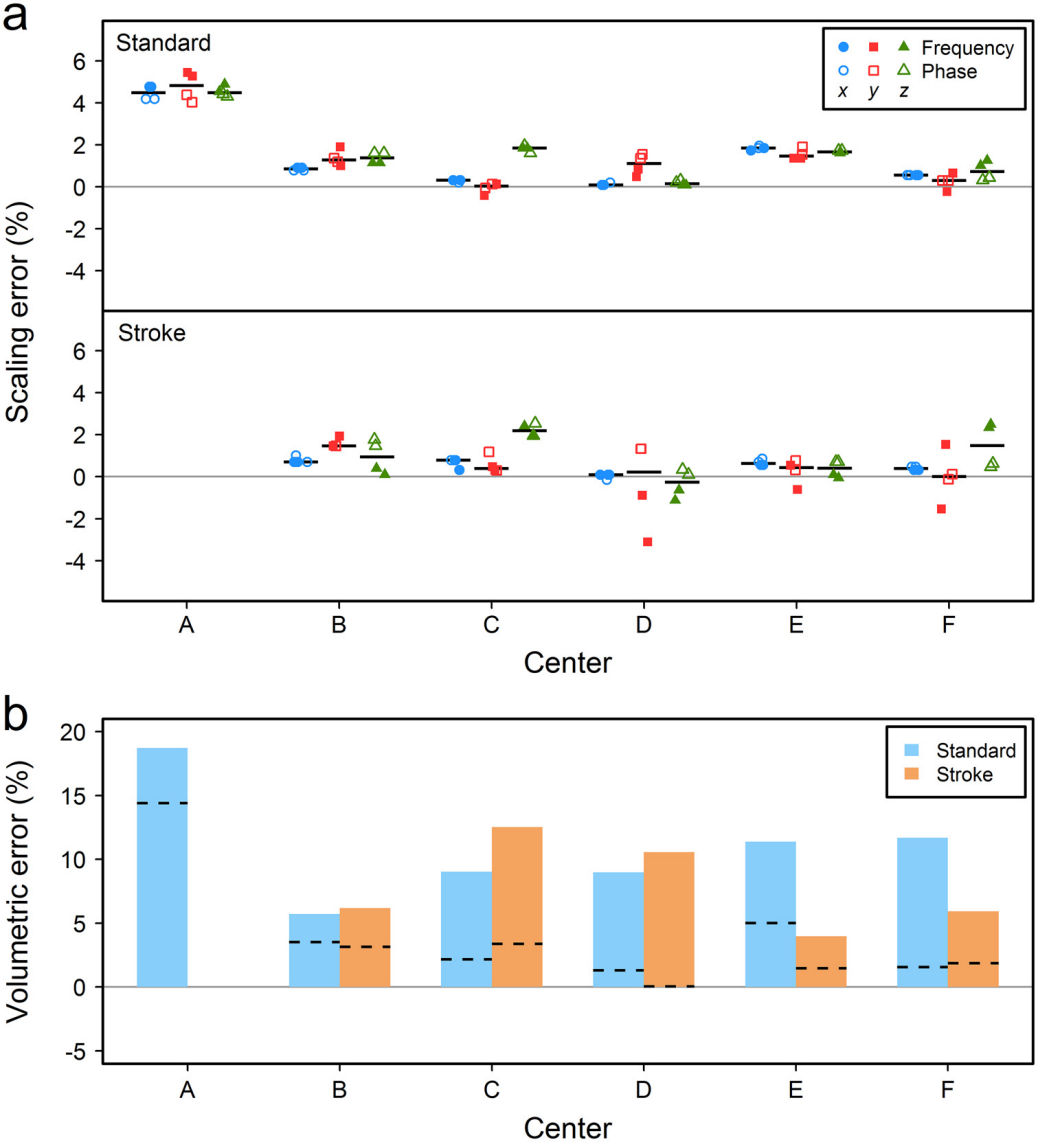
Between-scanner variability. (a) Percentage linear scaling error across the three orthogonal directions in the MRI systems with median values shown by the black dashes and (b) the percent volumetric error with values predicted by the median scaling errors shown by the dashed lines. Scaling errors are based on the internal dimensions of the whole phantom measured on scans in all imaging planes, while volumetric errors are based on the central frustum-shaped compartment segmented on axial scans. Only the standard protocol was used to evaluate scanner “A” as no stroke protocols were utilized at this center prior to our study.

**Fig 3:**
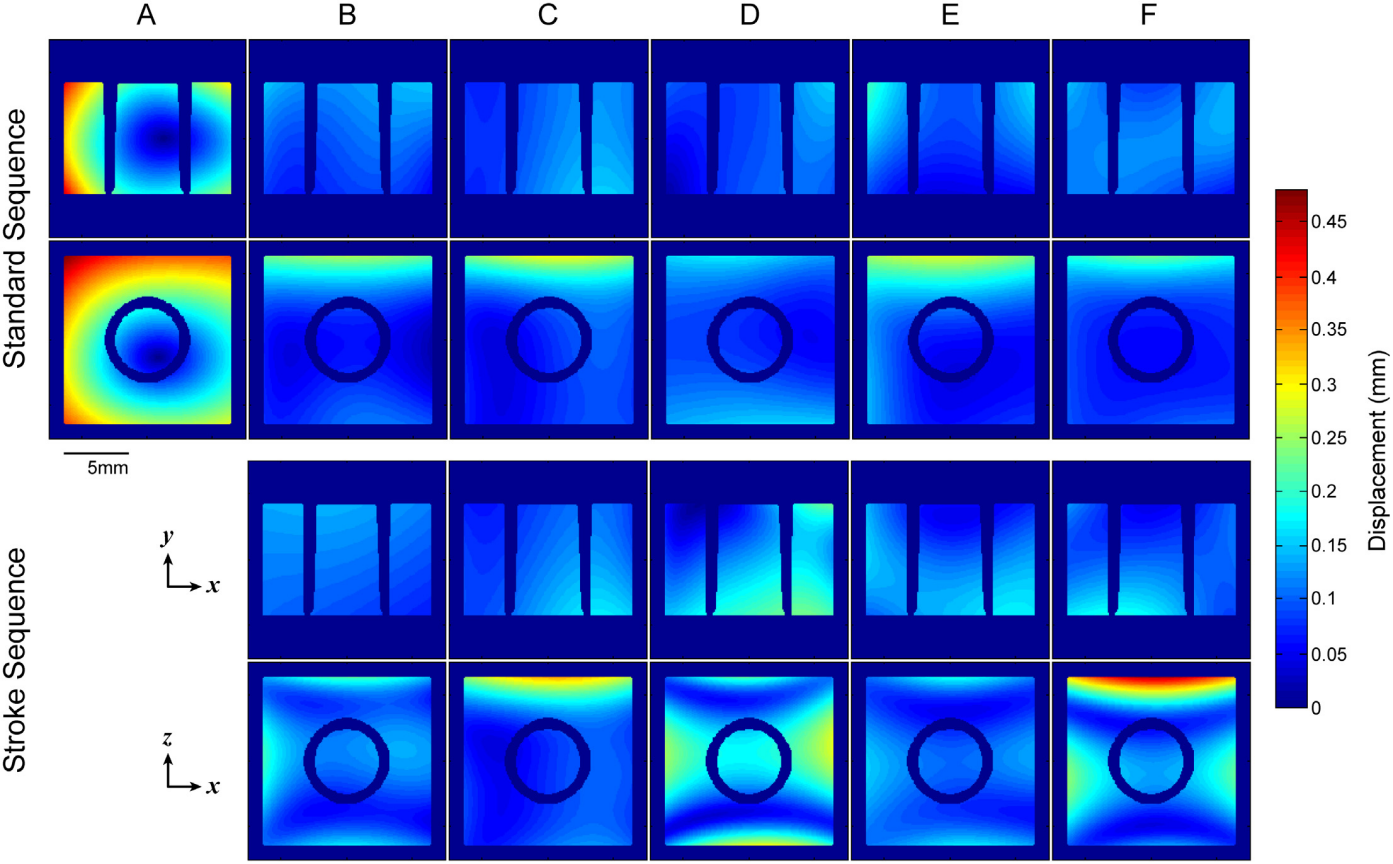
Deformation needed to recover reference CT scans from MRI data. The maps show the in-plane absolute Euclidean displacement required to recover the true shape of the phantom in axial (*x-y*) and coronal (*x-z*) MRI data from the between-scanner variability assessment. Phase encoding is in the horizontal direction (*x*) in both planes. It is evident that scanning using an identical (standard) sequence ensures better correspondence between images from scanners “B”-“F” in terms of distortion effects. Correspondence declined when stroke sequences “b”-“f” were used, particularly in the coronal plane. Furthermore, overall distortion was characterized by two differing patterns; images from system “A” were uniformly stretched in both directions, whereas minor non-linearities were present in images from systems “B”-“F”. It should be emphasized that while these deformation maps successfully demonstrate the overall distortion of the MRI data compared to reference images, they do not represent the true geometric distortion in the MRI systems; a phantom with a large number of equidistant control points (grid structure) is often required for this purpose. Corresponding MRI images are shown in S2 Fig.

Fig 2a gives the scaling errors for each of the six participating sites. Linear scaling errors were mostly positive (measured dimensions were larger than ground truth values) in all scanners for both standard and stroke sequences. Despite that the overall measurement accuracy in stroke sequences was not worse than the accuracy in the standard sequence, measurements were more dispersed. In systems “B”-“F” median errors per direction were within 0.03% to 1.84% and -0.27% to 2.19% for the standard and stroke sequences respectively. The standard sequence “a” alone was used to evaluate scanner “A”; this system overestimated the phantom’s dimensions by 4.47% across *x* and *z* directions and 4.82% across *y* direction (median values). Inspection of 2D deformation maps (Fig 3) and corresponding MRI images through the center of the phantom (S2 Fig) reveals that overall distortion is characterized by two differing patterns; images from scanner “A” have an almost isotropic expansion, whereas images from the rest of the scanners show the presence of minor non-linearities, particularly across phase encoding and predominantly in stroke sequences. Post hoc scanning in system “A” using in vivo sequence “b” and a modified version of this sequence which theoretically amplifies distortion effects did not produce pronounced non-linearities in this system (described in S1 Appendix and sample images shown in S3 Fig).

In accordance with linear scaling errors, the estimated volume was larger in system “A” (18.73% overestimation) compared to the rest of the scanners (range 5.71% to 11.67%, scanners “B” and “F” respectively) when the standard protocol was used (Fig 2b). Despite that stroke sequences had a better overall volumetric accuracy compared to the standard sequence (median error 6.19% versus 9.02% in scanners “B”-“F”), their measurement variability was higher, with errors ranging from 3.96% to 12.51% (scanners “E” and “C” respectively). Measured volumes for all systems and both sequences were higher than corresponding volumes predicted by linear scaling errors, with percentage differences between the two ranging from 2.19% to 10.10% for the standard sequence (scanners “B” and “F” respectively) and 2.49% to 10.53% for stroke sequences (scanners “E” and “D” respectively; Fig 2b). Finally, we observed that volumes measured using the standard sequence were often influenced by noise in the data (for example, the difference between measured and predicted volumes was low for scanner “B” and high for scanner “F” which had the highest and lowest level of noise respectively), while volume overestimation in stroke sequences was positively associated with slice thickness (for example, the difference between measured and predicted volumes was higher for centers “C” and “D” which utilize sequences with 1mm thick slices).

**Fig 4:**
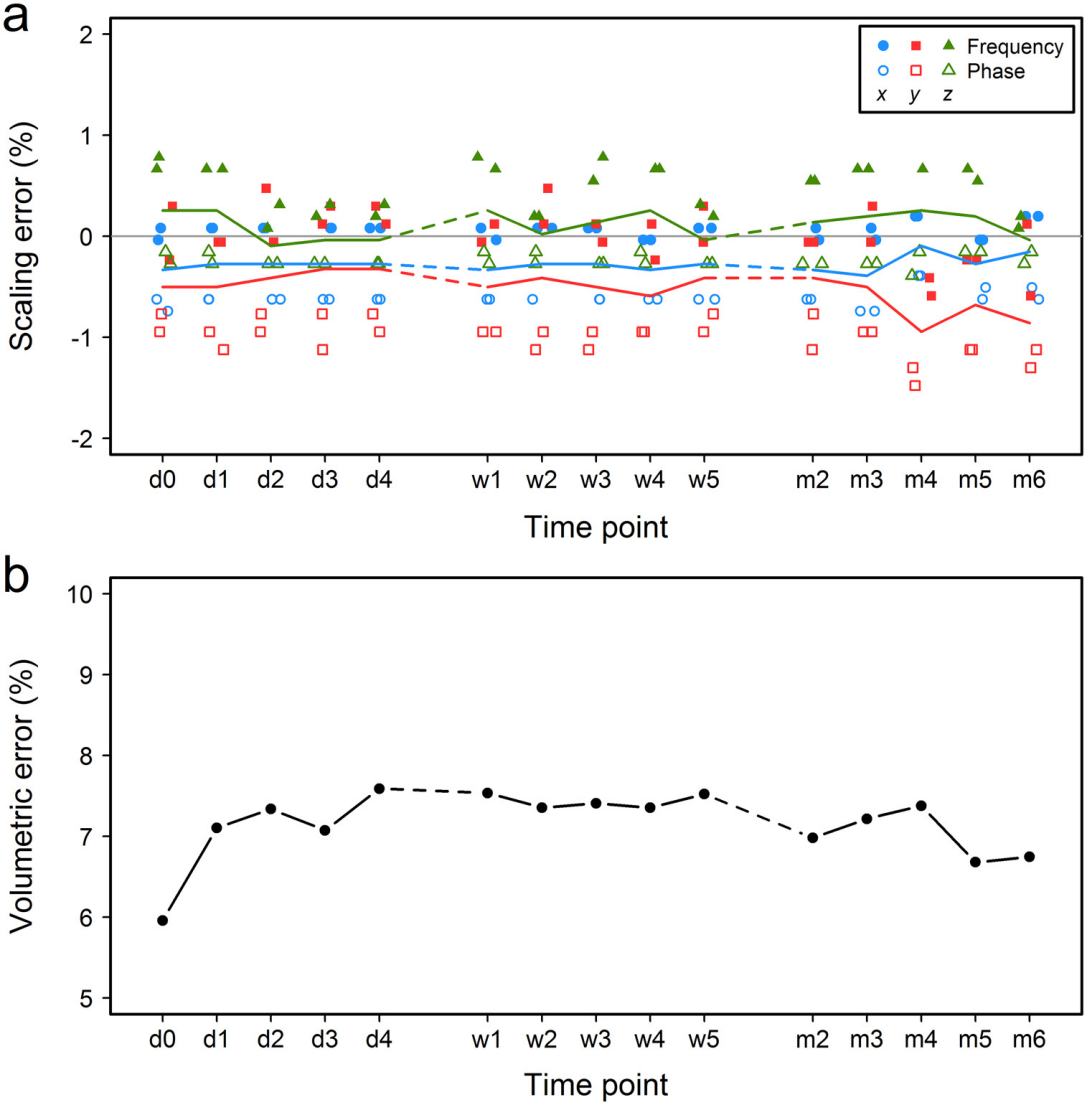
Within-scanner variability. (a) Percent linear scaling error across the three orthogonal directions and (b) the percent volumetric error measured in system “A” using the standard sequence “a” after calibration of the gradient coils. The colored lines in (a) follow the median values at each time point. d indicates day; w, week; m, month. “d0” is the baseline time point immediately after calibration.

Following between-scanner assessment, we calibrated the worst-performing system “A” based on measurements obtained with the standard sequence “a” and recorded drifts in its performance longitudinally over six months. Linear scaling errors were reduced significantly at baseline scanning (median values: -0.33% in *x* direction, -0.50% in *y* direction, and 0.25% in *z* direction; “d0” time point in Fig 4a). These figures did not change significantly during the subsequent longitudinal assessment, with median errors for all three directions and time points remaining within ±1% (Fig 4a). Fluctuation in the apparent size of the phantom was similar for all three imaging intervals (daily, weekly and monthly) across each of the three orthogonal directions, despite a seeming increase in variability during the monthly assessment in Fig 4a. However, there was a noticeable drift in the performance of the *y*-gradient from the second to the last month (median -0.41% to -0.86%, “m2” to “m6” time points respectively in Fig 4a). The volume of the phantom’s central compartment was overestimated at all time points (Fig 4b). Despite small differences in the variability in volumetric error between the three time intervals, the overall variance across the whole six month period was very small compared to between-scanner variability (median 7.33%, interquartile range (IQR) 0.43%).

Lastly, we did not observe any persistent differences in scaling errors between frequency and phase encoding directions in systems “B”-“F” (sample size was too small to perform any statistical comparisons), but in system “A” errors in frequency encoding direction were significantly different compared to errors in phase encoding (frequency: median 0.12%, IQR 0.35%; phase: median -0.63%, IQR 0.67%; Mann-Whitney U test: *U* = 136, *p*<0.001, based on all within-scanner variability data).

## Discussion

Despite that the use of preclinical MRI systems in experimental research was intensified long ago by the need of in vivo assessment of injury over extensive periods of time, individual centers still rely on annual maintenance of their systems that is performed at different times across centers [18], and hardware and protocols for in vivo imaging are highly heterogeneous between centers [9]. The effect of these in animal studies remained largely unexplored, but the variability in treatment efficacy based on MRI lesion volume can be profound, as recently suggested by findings of possibly the first multicenter preclinical stroke trial [19]. Influenced by such inferences, we successfully implemented a simple QA approach for evaluating geometric accuracy in several MRI systems as used for the same purpose and specifically the assessment of infarct size in rodent models of focal cerebral ischaemia. Our study found significant errors when miscalibrated gradient coils are used. To identify whether different sequences have the same variability as a standard one between centers, measurements taken with an identical sequence were compared to measurements taken with each center’s preferred in vivo sequence. We showed that an identical sequence ensures better agreement between centers and could thus be more favorable in a multicenter setting.

Linear scaling errors were estimated according to the internal dimensions of the whole phantom measured in scans in all imaging planes, while volumetric errors were based on the volume of the central compartment of the phantom measured in the axial plane (Fig 1). The axial plane was chosen for volume measurements as it corresponds to the coronal plane in rodent brain MRI often used in experimental studies and the phantom’s volume in this plane encompasses marked partial volume artefacts characteristic of biological structures. While acceptance limits for geometric accuracy in preclinical systems are currently not available, scaling errors in systems “B”-“F” were comparable and mostly within the ±1% limit proposed by the American College of Radiology for clinical systems [8]. Errors in system “A” were higher, agreeing with the observations of a previous study that assessed the accuracy of a scanner from the same vendor [18]. In addition, we identified a persistent difference in measurements between frequency and phase encoding directions in all imaging planes in this system, both before and after calibration. Linewidths from manual global magnetic field shimming were well below 100Hz for all scans and the receive bandwidth was set to 100kHz; these are often sufficient to negate most distortion effects. Yet, the presence of this difference points to other sequence limitations and MRI artefacts such as ghosting or Gibbs ringing due to Fourier transform imperfections in phase encoding (additional distortion causes are described later in this section).

In contrast, volumetric errors in all scanners were positive and rather inconsistent and volumetric variability presented dissimilar patterns in short- and long-term repeated scans. This emphasizes that standard volume quantification using 2D sequences can be greatly influenced by factors other than linear scaling errors and normal system performance drift. Firstly, a large voxel size can blur the boundary of an object due to partial volume effects, leading to a seeming boundary outward shift and thus volume overestimation. This is a well-known phenomenon that has been observed in previous QA studies [20, 21]. Here, stroke sequences “c” and “d” had the thickest slices and led to the two largest differences between measured and predicted volumes amongst centers, with “d” characterized by both the largest overall voxel size and largest difference. Sequences using thinner slices and therefore more slices through the volume of interest generally reduce this artefact, explaining the higher overall measurement accuracy of stroke sequences compared to the standard sequence in systems “B”-“F”. Secondly, while a high signal-to-noise ratio (SNR) is generally preferred as it improves image quality and volume of interest delineation, in slices with significant partial volume effects the apparent contrast of the volume of interest and background may be enhanced, causing inclusion of false positives. In this study, data from center “B” had a noticeably lower SNR compared to data from other centers, leading to small differences between measured and predicted volumetric errors. Lastly, volume estimation using 2D sequences can be highly dependent on slice positioning, particularly when the volume of interest is small or the slices are thick, and even small variations in slice positioning can introduce inconsistencies in partial volume and gradient distortion effects. In this study, phantom scanning adhered to detailed instructions for at least slice positioning and images were comparable, therefore any influence of this factor could have only been minimal. These remarks suggest that formulas currently used for estimating volumes from in vivo 2D MRI data could be modified to account for unwanted misestimations [22, 23], but further studies are needed to examine the usefulness of such approaches in individual study designs.

Subtle spatial distortion effects were observed in the acquired MRI data that may have further contributed to the increased variability in measured errors. We can attribute these to the following factors. Firstly, non-linearities in the gradient magnetic fields could be present due to possible design limitations of gradient coils in high-field systems, such as short bore or short gradient rise times that induce eddy currents in nearby conducting materials [24, 25]. This is supported by the observation that the distortion was similar for systems “B”-“F” making use of gradient coils from the same manufacturer, which differed from the pattern seen in system “A”. The non-linearities in systems “B”-“F” could not be observed in system “A”, even after post hoc scanning in this system using in vivo sequence “b” and a modified version of it which theoretically amplifies distortion effects. However, the phantom’s size is not sufficient to allow deduction of rational acceptance decisions (maximum permitted linearity over an 80mm diameter of spherical imaging volume is ±4–5%, as specified by manufacturers for these coil systems). Secondly, magnetic susceptibility differences along the interfaces of the phantom’s contrast solution, plastic and outside air may have produced local magnetic field inhomogeneities. Both plastic polymers and water are diamagnetic materials [26] and the characteristic susceptibility effect of focal regions with signal void and opposing regions with bright signal was not apparent along the plastic-solution interface in any of the acquired scans. However, the presence of paramagnetic air in close proximity might have been influential, particularly for systems “B”-“F”, suggesting that susceptibility effects could produce different artefacts in brain imaging between centers (the sinuses and nasal cavity lie close to the brain). In addition, a narrow receive bandwidth may introduce positional shifts in the frequency encoding direction as it relatively decreases gradient amplitude and enhances susceptibility artefacts. This could partially explain why non-linearities were more enhanced in images taken using the stroke sequences utilizing about half of the standard sequence’s receiver bandwidth in an effort to improve SNR. In general, geometric distortion in small animal scanners increases rapidly as the distance increases from the magnet’s isocenter, indicating that even relative measures, such as the lesion to brain size ratio, could be affected detrimentally. “Online” geometric distortion correction algorithms similar to those inherent in human MRI systems, or “offline” post-scanning correction methods should be used in preclinical imaging as well [18, 27–29].

For the purposes of this study, phantoms based on LEGO bricks were ideal for a number of reasons: 1) the sizes of the central volume of interest and the phantom itself are similar to those of a large subcortical infarct and the part of the rat brain often imaged in stroke respectively (12-13mm across the rostro-caudal direction in middle cerebral artery occlusion models); 2) their cuboid shape allows reproducible placement in the scanners and accurate measurement of dimensions in all directions, in contrast with spherical or cylindrical phantoms; 3) they are manufactured with superior precision than most current alternative approaches, such as 3D printing (20μm) [13]; 4) they are made of thermoplastic polymers with great impact strength, dimensional stability and MRI compatibility [12, 26]; and 5) they are very affordable and widely available. Recently, other larger and more complex phantom designs have been proposed for monitoring geometric accuracy or performing 3D geometric distortion correction in preclinical MRI [18, 29–31], but their size inhibits their use with various imaging coils, they generally have lower construction precision and are currently not as cost-effective; as such, they were not used in this multicenter study. LEGO bricks were also used successfully for the development of clinical phantoms before [25].

Imaging performance can be an important cause of lesion size variability, as suggested by our findings, but a plethora of other factors are often considered as primary contributors, including inconsistencies in surgery and stroke model induction, and inter-animal differences in cerebrovasculature and comorbidity. While heterogeneity in some aspects of animal experimentation might help in mimicking the complex human condition as much as possible [32], outcome assessment methods, and in particular MRI, can introduce a moderate but systematic bias in measurements if not properly calibrated or carried out; standardization is therefore essential. In single-center studies where inferences are often based on relative measurements and comparisons against similarly assessed controls, MRI accuracy may not be of critical importance. Yet, in a multicenter setting absolute measurements are primarily combined and the number of animals per group and per center can be highly variable, thus the use of the modality must be both accurate and equivalent between centers. Unfortunately, pulse sequences and hardware that eliminate all the aforementioned distortions are currently not available for in vivo MRI experiments which require very fast scanning. However, we believe that some actions to reduce variability between scanners and allow effective comparison of data can easily be taken. We suggest that pulse sequences should be standardized at least in terms of voxel size (matrix size, FOV, slice thickness) and slice positioning relative to an anatomical feature (for example, the bregma or the rostral end of the rhinal fissure in rodent brain MRI). As long as lesions are large enough in the set FOV, sequences should make use of thin slices to minimize partial volume effects and compromise in-plane resolution to preserve SNR. Scanners with the same field strength and similar RF coil designs should be used were possible. To further accommodate homogeneity in SNR due to hardware differences, other parameters can be adjusted accordingly (TR, TE, number of averages, receive bandwidth etc.). Finally, a QA protocol using an identical phantom, a standardized in vivo pulse sequence and basic data analysis methodology, such as the one implemented here, should be applied before the start of the study at each center to improve the performance and comparability of the systems. As suggested by our findings and those of others [18], longitudinal studies can be performed efficiently over a period of at least 6 months following system calibration as the measured drift in phantom volume (0.4% based on the reported median and IQR of error) is much smaller than the expected biological change in ischemic injuries (can be 50% or more), assuming that no changes in scanner software and equipment take place within the duration of the experiment. Where the longitudinal variability of more stable anatomical regions is to be evaluated, such as brain size, assessment of geometric accuracy should ideally be performed on a weekly basis [8]. Procedures such as those described here must be considered the minimum set of QA tests performed. Additional tests may be needed if scanners are used routinely for advanced stroke MRI, such as diffusion-weighted and functional MRI that place even higher demands on system performance. Characterization of the effects of these imaging methods across centers was beyond the scope of this study, but should be the focus of future work for the establishment of a comprehensive multicenter QA program.

A major limitation of this study is that the small size of the phantom precludes its use for monitoring geometric accuracy in studies assessing structural biomarkers larger than those in models of neurological diseases in rodents. Other approaches [18, 29, 30] or larger LEGO bricks can be used for this purpose. In addition, while the phantom has similarities with a rat brain in terms of overall size and T_2_ relaxation properties, the volume of interest in this study does not mimic the shape of an actual brain injury, or the contrast in intensities between the injured and normal tissue. Methods for creating very small and stable structures with lesion-like characteristics are currently not available; even so, the phantom constitutes a useful method for examining the influence of scanning parameters and identifying sources of variability in small animal MRI. A further limitation is that, due to logistical reasons, only 7T scanners were included in this study and longitudinal scanning was performed with the only Agilent^®^ scanner used in participating centers. Testing of more preclinical systems would have provided more conclusive evidence for the role of field strength and vendor in geometric accuracy. Finally, small deviations from the prescribed scanning instructions sent to each preclinical center may have introduced variation in some of the measurements that could possibly render corresponding comparisons less effective. Nevertheless, these deviations would be representative of the true heterogeneity in experimental imaging and the subsequent variation in measured biological effects.

## Conclusions

The study uncovers a widespread inconsistency in geometric accuracy of various preclinical MRI scanners, raising concerns regarding the comparability of measured outcomes across centers. Significant errors in measurements can be present when miscalibrated MRI gradient coil systems are used. Scanners of the same manufacturer have largely similar performance and imaging using an identical sequence ensures better agreement between measurements. However, the impact of the observed errors on actual animal data should be examined further before standard protocols are devised for use in collaborative studies. We hope that this study will promote development and standardization of methods for routine scanner QA and in vivo imaging, similar in rigor to those utilized in clinical centers. This will be pivotal for effective pooling of data and derivation of valid statistical inferences in future multicenter animal studies.

## Supporting Information

S1 Appendix Supporting materials and methods. (DOCX)

S1 Fig Bland-Altman plots validating the performance of the semi-automated analysis tool. The measurements from the first manual analysis by XM were used as the subtrahends for estimating the differences in all comparisons. The solid blue line in each plot indicates the mean difference (representing accuracy; value given in each plot) and the dashed blue lines the 95% limits of agreement (mean±1.96 standard deviations of the difference). The accuracy of the semi-automated analysis is high and the dispersion of differences in the manual versus semi-automated analysis is similar to the intra- and inter-observed comparisons, indicating excellent performance by the semi-automated tool. A great overlap between volumes segmented manually and semi-automatically was found (Dice coefficient: median = 0.982, IQR = 0.975–0.983).
(TIF)

S2 Fig Sample images from all evaluated systems. These are slices through the center of the phantom in the axial (*x-y*) and coronal (*x-z*) planes. Only the standard sequence “a” was used to evaluate system “A”. Phase encoding is in the horizontal direction (*x*) in both planes. Images taken using the stroke sequences for scanners “B”, “D”, “E”, and “F” were scaled to match the in-plane resolution of the standard sequence for direct comparison. The figure shows that images acquired using scanner “A” were characterized by a rather isotropic expansion, whereas images taken using systems “B”-“F” had minor non-linearities, particularly those acquired using stroke sequences (examples indicated by the red arrows). Corresponding color maps visualizing the deformation required to recover the true shape of the phantom are shown in Fig 3 of the main article.
(TIF)

S3 Fig Influence of magnetic susceptibility effects on geometric distortion. Sample slices through the phantom in the axial (*x-y*) and coronal (*x-z*) planes are shown, acquired using system “A” (Table 1 of the main article) and three different sequences comprising various combinations of echo times and receive bandwidths. Images taken using sequences “b” and “b_modified_” were scaled to match the in-plane resolution of the standard sequence “a” for direct comparison. In contrast with the performance of systems “B”-“F” for different sequences (Fig 3 of the main article, S2 Fig), the overall shape of the phantom in this system was similar for all sequences, including “b_modified_” which comprises of an abnormally long echo time (93ms) and narrow bandwidth (40.3kHz). This suggests that magnetic susceptibility artefacts alone are not sufficient to describe the observed non-linearities in systems “B”-“F”, and that other system-related effects could be prevailing. To note, the images have improved intensity uniformity compared to images from the same scanner shown in S2 Fig, as they were taken following maintenance of the system and imaging coils.
(TIF)

S1 File Between- and within-scanner variability study data. (XLSX)
