# Supplementary material for: Multicenter Evaluation of Geometric Accuracy of MRI Protocols Used in Experimental Stroke
Source: PLoS One. 2016 Sep 7;11(9):e0162545. doi: 10.1371/journal.pone.0162545 (PMC5014410; doi:10.1371/journal.pone.0162545)
Supplement: S1 Appendix — (DOCX) [file pone.0162545.s001.docx]

**Supporting Materials and Methods**

**Use and Validation of the Semi-Automated Image Analysis Tool**

The tool uses the Canny edge detection technique [[1](#_ENREF_1)] in combination with basic morphological processing functions in MATLAB^®^ (2015a, The MathWorks Inc., Natick, Massachusetts, USA) to estimate the internal dimensions of the whole phantom and the volume of its central frustum-shaped compartment (Fig 1 of the main article). Initially, the tool automatically rotates the loaded dataset if the phantom is misaligned. Then, the boundary of the phantom is detected using Canny’s method [[1](#_ENREF_1)]. This technique was chosen as it detects edges by calculating local intensity gradients in the images; it is thus not affected by global intensity inhomogeneities that often confound thresholding techniques, such as various bias field patterns produced by different types of imaging coils. The internal dimensions of the phantom are measured in scans in all imaging planes by performing measurements at four different locations across opposing edges per direction (horizontal and vertical). The measurements are of subpixel accuracy, meaning that edges are detected at a finer resolution than that of the original image. The tool performs this by resizing the images before edge detection using bicubic interpolation, by a factor that can be chosen by the tool’s user; here, a factor of five was used, thus measurements had an accuracy five times better than the original pixel size. Larger scaling factors were not used as they could compromise the speed and robustness of analysis. This process is repeated in several slices and the mode value is calculated for each direction, thus discarding any abnormal measurements attributed to the phantom’s orientation marker or other artefacts in the images. The volume of the phantom’s central compartment is measured in axial scans by automatically identifying it in all slices, counting included pixels and multiplying by the voxel size to yield the total volume. Analysis takes about than 10 seconds per scan to perform. The tool, along with instructions for its use, is freely available to download from GitHub (<https://github.com/Edinburgh-Imaging/PreclinicalMRIPhantomAnalysis>).

The tool’s accuracy for estimating dimensions was evaluated by comparison with manual analysis of a dataset of 60 scans in all imaging planes and its accuracy for estimating volumes by comparison with manual segmentation of 10 scans in the axial plane. These scans simulate variations in scaling, location within the field of view and signal-to-noise ratio by adding Rician noise or smoothing with median filters. Manual measurements were performed in ImageJ (1.50b, Rasband, W.S., National Institutes of Health, Bethesda, Maryland, USA, <http://imagej.nih.gov/ij/>) twice by a physicist (XM) and once by an MRI technologist (RJL), each with at least 3 years of image analysis experience for intra- and inter-observer variability assessment. Statistical analysis was performed using Bland-Altman plots [[2](#_ENREF_2)] (S1 Fig). To evaluate the spatial overlap between manually and semi-automatically segmented volumes the Dice coefficient [[3](#_ENREF_3)] was estimated using custom-written code in MATLAB (results in S1 Fig label).

**Influence of Magnetic Susceptibility Effects on Geometric Distortion**

To examine whether geometric distortion is dependent on susceptibility-induced artefacts alone, post hoc phantom scanning using the calibrated Agilent Technologies^®^ 7T scanner (“A” in Table 1 of the main article) and three different sequences was performed: standard sequence “a” and in vivo stroke sequence “b” as described in Table 2 of the main article, and a modified version of “b”; this “b_modified_” sequence theoretically amplifies magnetic susceptibility effects by using an increased effective echo time (93ms) and a decreased receive bandwidth (40.3kHz). Other parameters of “b_modified_” matched the parameters of “b”. Sample images are shown in S3 Fig.

**References**

1. Canny J. A Computational Approach to Edge Detection. Pattern Analysis and Machine Intelligence, IEEE Transactions on. 1986;PAMI-8(6):679-98. doi: 10.1109/TPAMI.1986.4767851.

2. Martin Bland J, Altman D. STATISTICAL METHODS FOR ASSESSING AGREEMENT BETWEEN TWO METHODS OF CLINICAL MEASUREMENT. The Lancet. 1986;327(8476):307-10.

3. Dice LR. Measures of the Amount of Ecologic Association Between Species. Ecology. 1945;26(3):297-302. doi: 10.2307/1932409.
