# Supplementary figures and images for: Multicenter Evaluation of Geometric Accuracy of MRI Protocols Used in Experimental Stroke
Source: PLoS One. 2016 Sep 7;11(9):e0162545. doi: 10.1371/journal.pone.0162545 (PMC5014410; doi:10.1371/journal.pone.0162545)

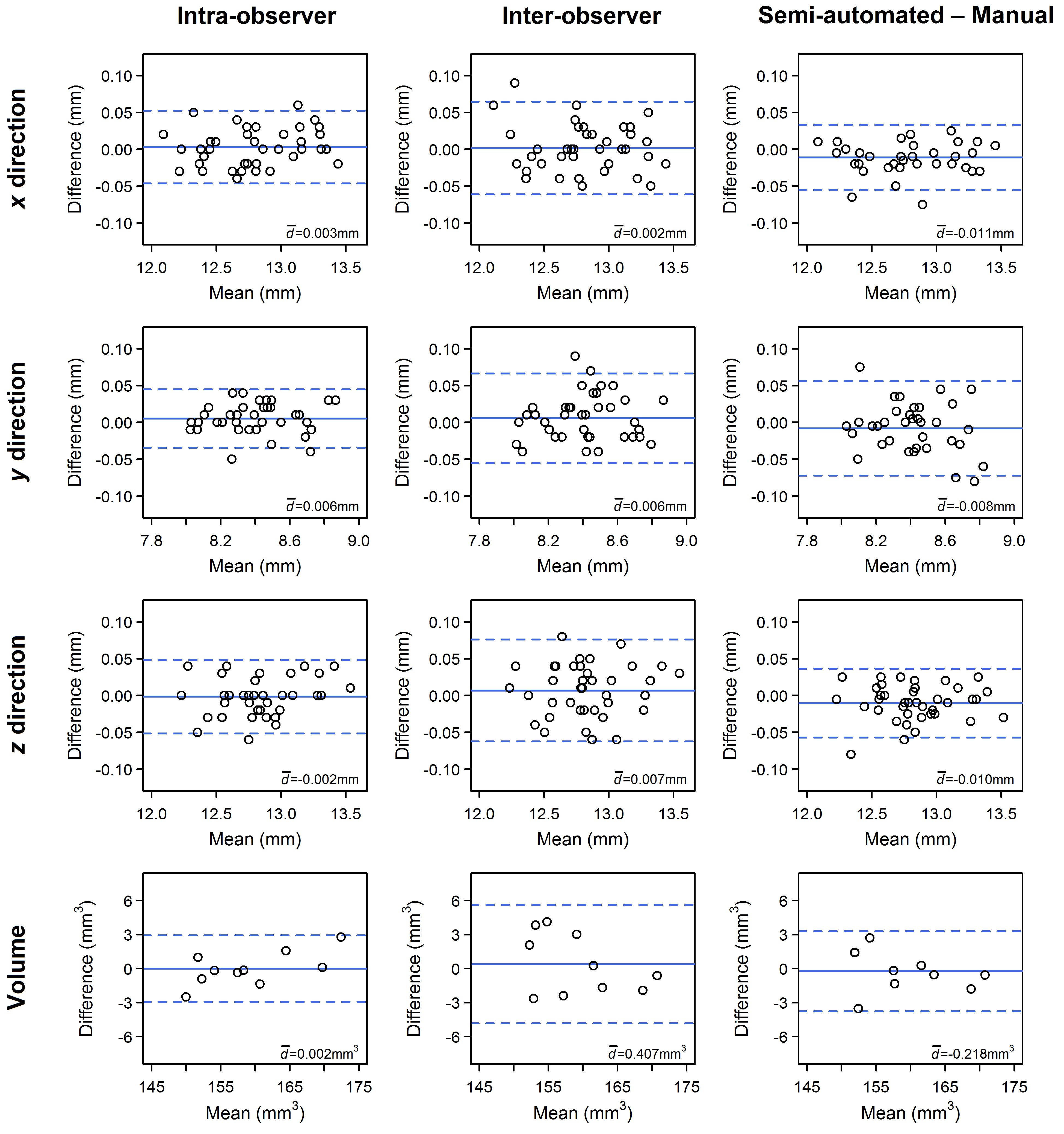

Supplement: S1 Fig — The measurements from the first manual analysis by XM were used as the subtrahends for estimating the differences in all comparisons. The solid blue line in each plot indicates the mean difference (representing accuracy; value given in each plot) and the dashed blue lines the 95% limits of agreement (mean±1.96 standard deviations of the difference). The accuracy of the semi-automated analysis is high and the dispersion of differences in the manual versus semi-automated analysis is similar to the intra- and inter-observed comparisons, indicating excellent performance by the semi-automated tool. A great overlap between volumes segmented manually and semi-automatically was found (Dice coefficient: median = 0.982, IQR = 0.975–0.983). (TIF) [file pone.0162545.s002.tif]

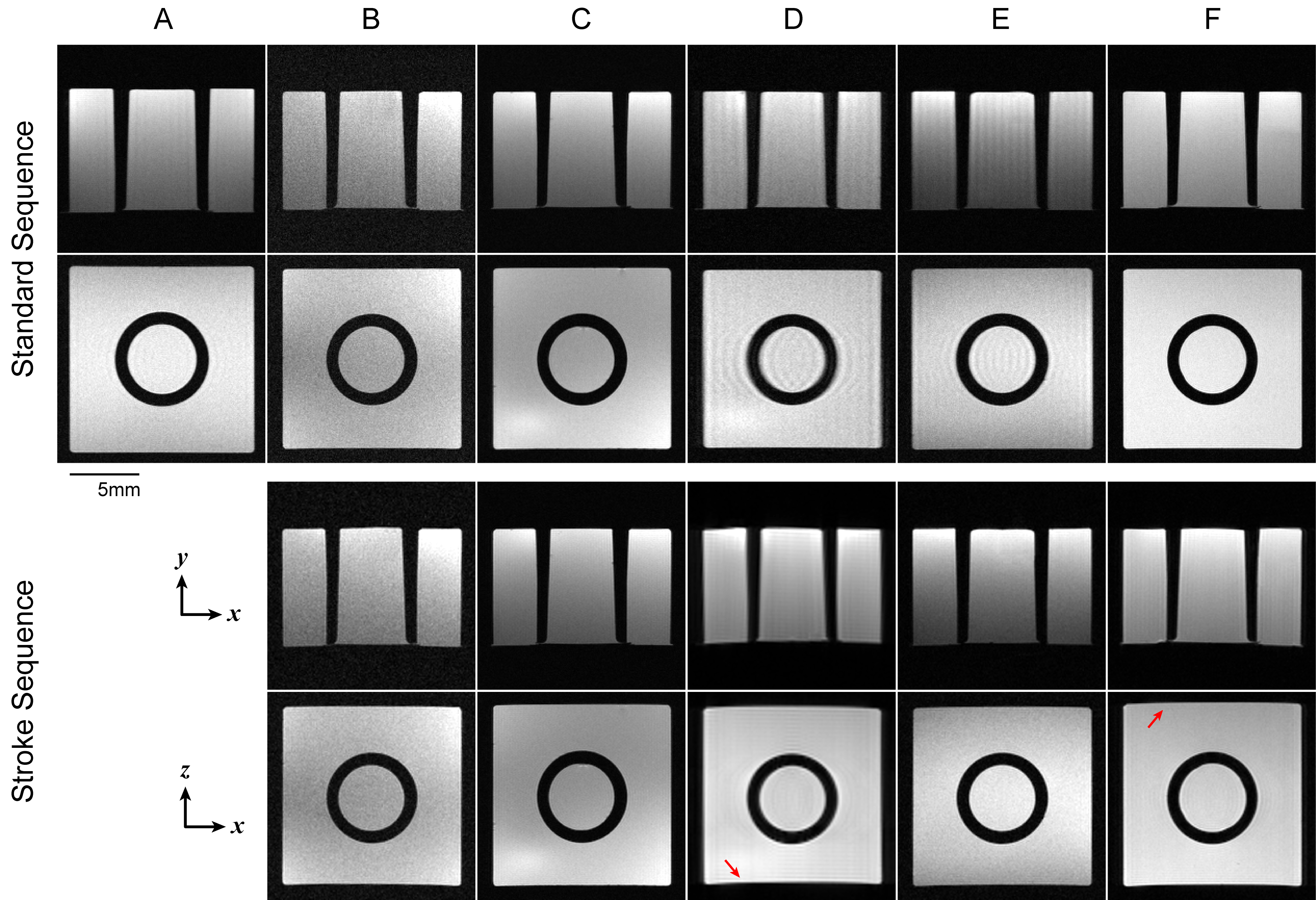

Supplement: S2 Fig — These are slices through the center of the phantom in the axial (x-y) and coronal (x-z) planes. Only the standard sequence “a” was used to evaluate system “A”. Phase encoding is in the horizontal direction (x) in both planes. Images taken using the stroke sequences for scanners “B”, “D”, “E”, and “F” were scaled to match the in-plane resolution of the standard sequence for direct comparison. The figure shows that images acquired using scanner “A” were characterized by a rather isotropic expansion, whereas images taken using systems “B”-“F” had minor non-linearities, particularly those acquired using stroke sequences (examples indicated by the red arrows). Corresponding color maps visualizing the deformation required to recover the true shape of the phantom are shown in Fig 3 of the main article. (TIF) [file pone.0162545.s003.tif]

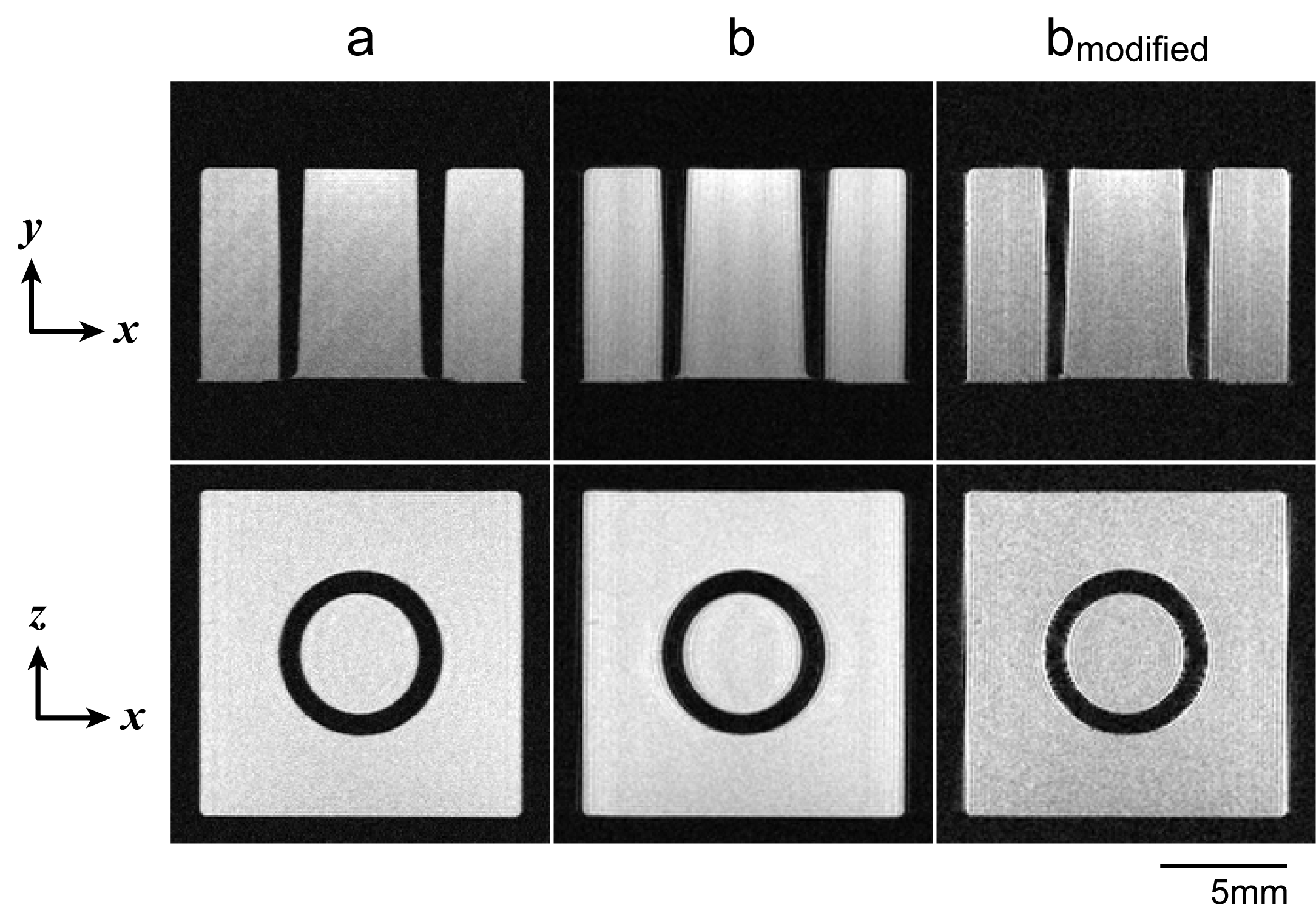

Supplement: S3 Fig — Sample slices through the phantom in the axial (x-y) and coronal (x-z) planes are shown, acquired using system “A” (Table 1 of the main article) and three different sequences comprising various combinations of echo times and receive bandwidths. Images taken using sequences “b” and “bmodified” were scaled to match the in-plane resolution of the standard sequence “a” for direct comparison. In contrast with the performance of systems “B”-“F” for different sequences (Fig 3 of the main article, S2 Fig), the overall shape of the phantom in this system was similar for all sequences, including “bmodified” which comprises of an abnormally long echo time (93ms) and narrow bandwidth (40.3kHz). This suggests that magnetic susceptibility artefacts alone are not sufficient to describe the observed non-linearities in systems “B”-“F”, and that other system-related effects could be prevailing. To note, the images have improved intensity uniformity compared to images from the same scanner shown in S2 Fig, as they were taken following maintenance of the system and imaging coils. (TIF) [file pone.0162545.s004.tif]
